# Correlation of Blood Lipid and Serum Inflammatory Factor Levels With Hypertensive Disorder Complicating Pregnancy

**DOI:** 10.3389/fsurg.2022.917458

**Published:** 2022-06-17

**Authors:** Wangxiang Chen, Yan Guo, Xia Yao, Di Zhao

**Affiliations:** ^1^ Department of obstetrics, Heji Hospital Affiliated to Changzhi Medical College, Changzhi, China; ^2^ Department of obstetrics, Heping Hospital Affiliated to Changzhi Medical College, Changzhi, China

**Keywords:** hypertensive disorder complicating pregnancy, blood lipid, inflammatory factors, preeclampsia, predictive value

## Abstract

**Purpose:**

To explore the changes of blood lipid and serum inflammatory factors in pregnant women with hypertensive disorder complicating pregnancy (HDP) and the relationship with disease development.

**Methods:**

107 pregnant women with HDP who had regular prenatal examination in our hospital from July 2018 to July 2021 were selected as the research objects. According to the severity of the disease, they were divided into gestational hypertension group, mild preeclampsia group and severe preeclampsia group. 30 healthy pregnant women who underwent prenatal examination in the same period were selected as the healthy group. Serum total cholesterol (TC), triglyceride (TG), high density lipoprotein cholesterol (HDL-C), low density lipoprotein cholesterol (LDL-C), lipoprotein-associated phospholipaseA2 (Lp-PLA2), C- reactive protein (CRP), interleukin -6 (IL-6), tumor necrosis factor-α (TNF-α) were measured. Receiver operating characteristic curve (ROC) was used to analyze the predictive value of blood lipid and serum inflammatory factors in pregnant women with HDP.

**Results:**

The levels of serum TC, TG and LDL-C increased with the progression of HDP, the level of serum HDL-C decreased with the progression of HDP (*P* < 0.05). The levels of serum Lp-PLA2, CRP, IL-6 and TNF-α increased with the progression of HDP (*P* < 0.05). The AUC of serum TC, TG, HDL-C and LDL-C levels for predicting HDP were 0.759, 0.854, 0.770 and 0.785, respectively. The AUC of serum Lp-PLA2, CRP, IL-6 and TNF-α levels for predicting HDP were 0.873, 0.991, 0.966 and 0.999, respectively.

**Conclusion:**

The levels of blood lipid and serum inflammatory factor are closely related to HDP, which has certain value in predicting the occurrence and development of HDP.

## Introduction

Hypertensive disorder complicating pregnancy (HDP) is the main cause of adverse maternal and infant outcomes, with an incidence of 5%–12%, and usually occurring after the 20th week of pregnancy (1). The main typical manifestations of HDP are hypertension, edema and proteinuria, but most pregnant women with mild HDP lack obvious clinical manifestations. Severe HDP can cause coma, convulsion, organ failure and other symptoms, and even lead to complications such as placental abruption, intrauterine fetal death and cerebrovascular events (2, 3). The pathogenesis and etiology of HDP have not been completely clear, and it is generally believed to be related to maternal, placental, fetal and other pathogenic factors (4). According to the severity of the disease, HDP can be divided into gestational hypertension, preeclampsia, eclampsia, pregnancy complicated with chronic hypertension and chronic hypertension complicated with preeclampsia. In HDP, gestational hypertension and preeclampsia are more likely to occur, which may induce maternal and infant death. Therefore, finding effective indicators for early prediction of the occurrence and development of HDP has attracted more and more attention. Studies have shown that the levels of blood lipids and serum inflammatory factors in pregnant women with HDP are often abnormally expressed (5). The purpose of this study is to observe the expression of blood lipid and serum inflammatory factors in pregnant women with HDP with different conditions, in order to carry out early intervention for pregnant women with HDP.

## Materials and Methods

### Research Object

107 pregnant women with HDP who had regular prenatal examination in our hospital from July 2018 to July 2021 were selected as the research objects. According to the severity of the disease (6), they were divided into gestational hypertension group (42 cases: There was no history of hypertension, and blood pressure began to rise to systolic blood pressure ≥140 mmHg or diastolic blood pressure ≥90 mmHg after 20 weeks of gestation), mild preeclampsia group (36 cases: The systolic blood pressure ≥140 mmHg or diastolic blood pressure ≥90 mmHg, and the 24 h proteinuria ≥0.3 g were measured more than twice) and severe preeclampsia group (29 cases: The systolic blood pressure ≥160 mm Hg and/or diastolic blood pressure ≥110 mmHg, and 24 h proteinuria ≥2.0 g or serum creatinine ≥1.2 mg/dL). 30 healthy pregnant women who underwent prenatal examination in the same period were selected as the healthy group. Inclusion criteria: Age ≥20 years old; Gestational week ≥20 weeks; Natural conception; Clear consciousness. Exclusion criteria: With essential hypertension; Multiple pregnancy; Non-assisted reproductive technology conception; The patients have taken drugs that affect lipid metabolism and inflammatory response; Combined diabetes; Complicated with heart disease; There is a history of alcoholism (Drinking more than 14 units of alcohol per week on average, 1 unit = 360 mL beer) and smoking (Smoking more than 1 cigarette per day).

### Research Methods

All pregnant women were taken 5 mL of venous blood in the morning fasting state. After anticoagulation and centrifugation, the serum was separated and stored at −70°C for testing. Researchers should do a good job in quality control, require the samples to be tested under the same conditions, and avoid the influence of interference factors on the test results. ① Blood lipid: Serum total cholesterol (TC) and triglyceride (TG) were measured by phosphoglycerol oxidase method, and high density lipoprotein cholesterol (HDL-C) and low density lipoprotein cholesterol (LDL-C) were measured by direct method. ② Serum inflammatory factors: Serum lipoprotein-associated phospholipaseA2 (Lp-PLA2) was measured by enzymatic kinetic method, serum C-reactive protein (CRP) was measured by latex enhanced transmission turbidimetry, and serum interleukin -6(IL-6) and tumor necrosis factor-α (TNF-α) were measured by enzyme-linked immunosorbent assay.

### Statistical Methods

SPSS 22.0 was used to analyze the data. Counting data was expressed by ratio, compared by *χ*^2^ test. Measurement data was expressed by mean ± standard deviation, compared by variance analysis. The predicted value was expressed by the ROC area under curve (AUC). The evaluation range of AUC: 0.5–0.7 indicates low predictive value, 0.7–0.9 indicates medium predictive value, ≥0.9 indicates high predictive value. *P* < 0.05 was statistically significant.

## Results

### Comparison of General Data in Each Group

There was no significant difference in age, gestational week, pregnancy times, pre-pregnancy body mass index and parity among the groups (*P* > 0.05) (Table 1).

**Table 1:** Comparison of general data in each group (*n*, %, $\bar{x}\pm s$).

Group	Number of cases	Age (years)	Gestational week (week)	Pregnancy times (times)	Pre-pregnancy body mass index (kg/m^2^)	Parity	
						Primipara	Multipara
Healthy group	30	28.48 ± 2.17	24.85 ± 1.24	2.83 ± 0.54	25.26 ± 2.71	21(70.00%)	9(30.00%)
Gestational hypertension group	42	28.29 ± 2.25	25.21 ± 1.06	2.79 ± 0.61	24.98 ± 2.63	29(69.05%)	13(30.95%)
Mild preeclampsia group	36	29.06 ± 2.13	24.92 ± 1.35	2.65 ± 0.60	25.18 ± 2.59	26(72.22%)	10(27.78%)
Severe preeclampsia group	29	28.43 ± 2.24	25.27 ± 1.13	2.72 ± 0.58	24.87 ± 2.61	17(58.62%)	12(41.38%)
*F/χ*^2^ value		0.877	0.989	0.624	0.145	1.540	
*P-*value		0.454	0.400	0.600	0.932	0.673	

### Comparison of Blood Lipid Levels in Each Group

**Figure 1:**
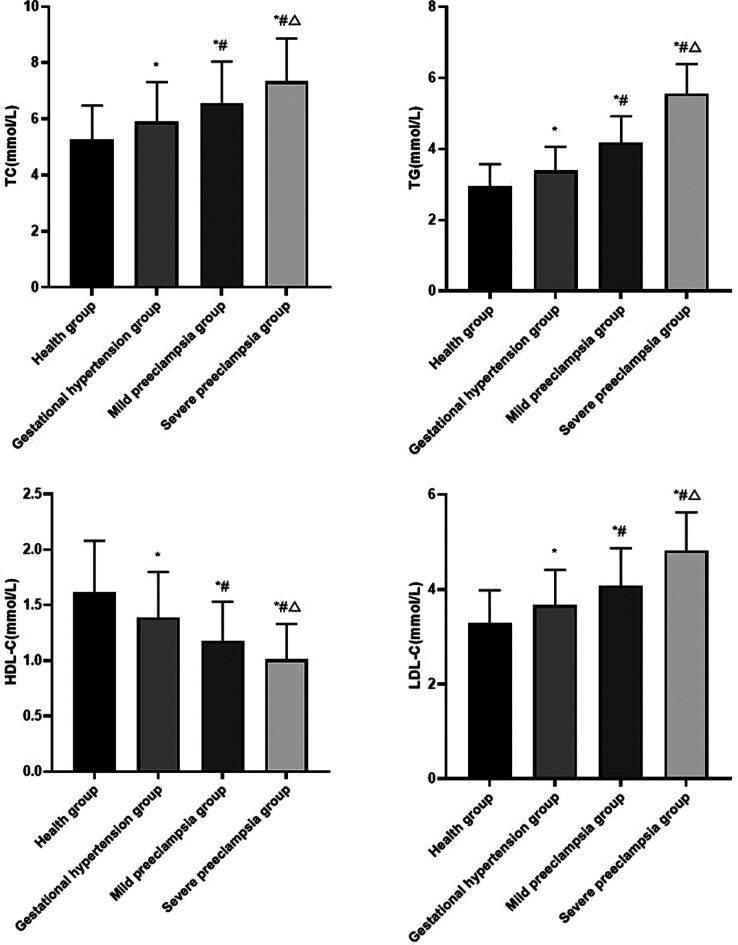
Comparison of blood lipid levels in each group Compared with healthy group, *P < 0.05; Compared with gestational hypertension group, #*P* < 0.05; Compared with mild preeclampsia group, △*P* < 0.05.

Compared with healthy group, the levels of serum TC, TG, LDL-C in gestational hypertension group, mild preeclampsia group and severe preeclampsia group all increased, while the level of serum HDL-C decreased. The levels of serum TC, TG and LDL-C increased with the progression of HDP, the level of serum HDL-C decreased with the progression of HDP (*P* < 0.05) (Figure 1).

### Comparison of Serum Inflammatory Factors in Each Group

**Figure 2:**
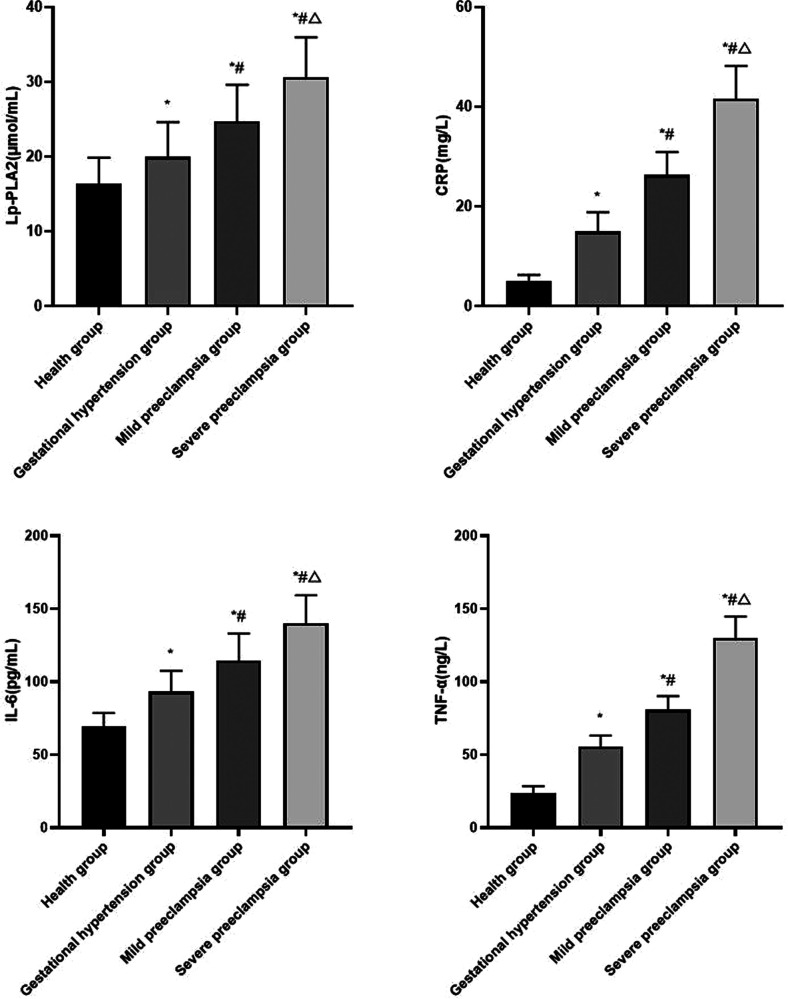
Comparison of serum inflammatory factors in each group Compared with healthy group, *P < 0.05; Compared with gestational hypertension group, ^#^*P* < 0.05; Compared with mild preeclampsia group, △*P* < 0.05.

Compared with healthy group, the levels of serum Lp-PLA2, CRP, IL-6 and TNF-α in gestational hypertension group, mild preeclampsia group and severe preeclampsia group all increased. The levels of serum Lp-PLA2, CRP, IL-6 and TNF-α increased with the progression of HDP (*P* < 0.05) (Figure 2).

### Predictive Value of Blood Lipid Level in Pregnant Women With HDP

**Figure 3:**
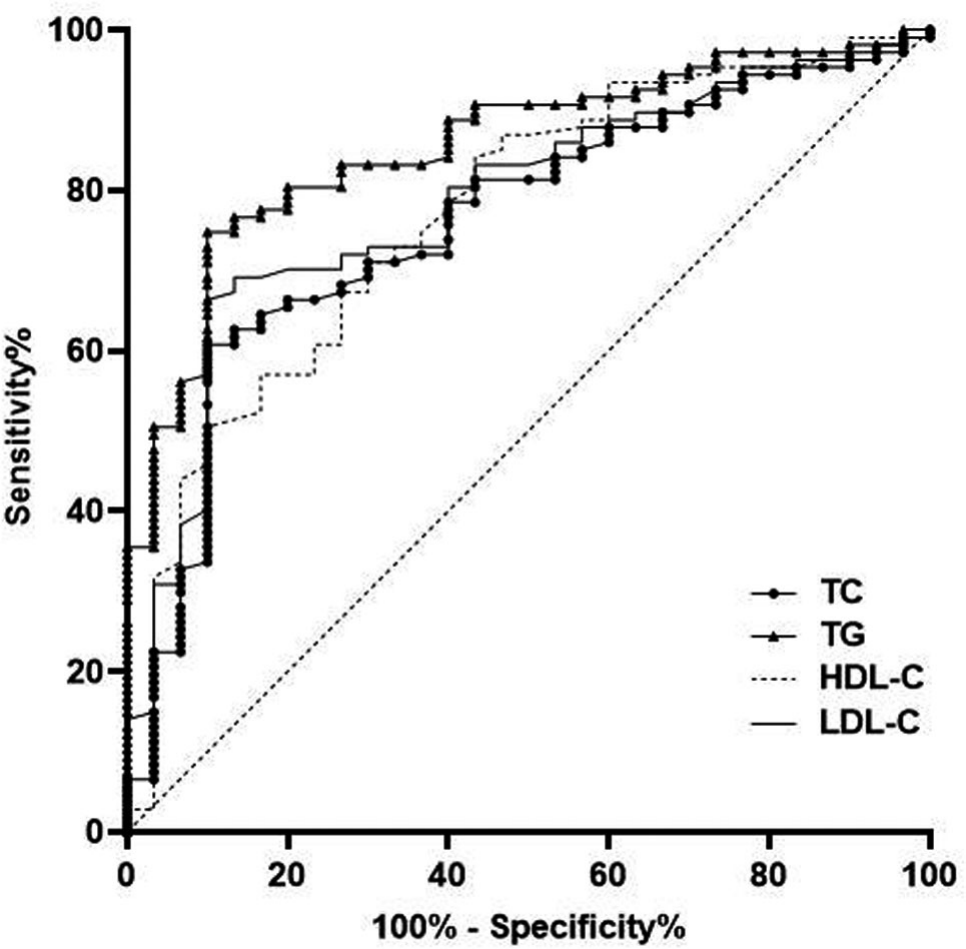
Predictive value of blood lipid level in pregnant women with HDP.

The AUC of serum TC, TG, HDL-C and LDL-C levels for predicting HDP were 0.759, 0.854, 0.770 and 0.785, respectively (Table 2 and Figure 3).

**Table 2:** Predictive value of blood lipid level in pregnant women with HDP.

Index	AUC	95% CI		Std. Error	*P*-value	Youden index
		Lower limit	Upper limit			
TC	0.759	0.666	0.853	0.047	<0.0001	0.507
TG	0.854	0.786	0.923	0.035	<0.0001	0.648
HDL-C	0.770	0.675	0.864	0.048	<0.0001	0.410
LDL-C	0.785	0.698	0.872	0.044	<0.0001	0.563

### Predictive Value of Serum Inflammatory Factors in Pregnant Women With HDP

**Figure 4:**
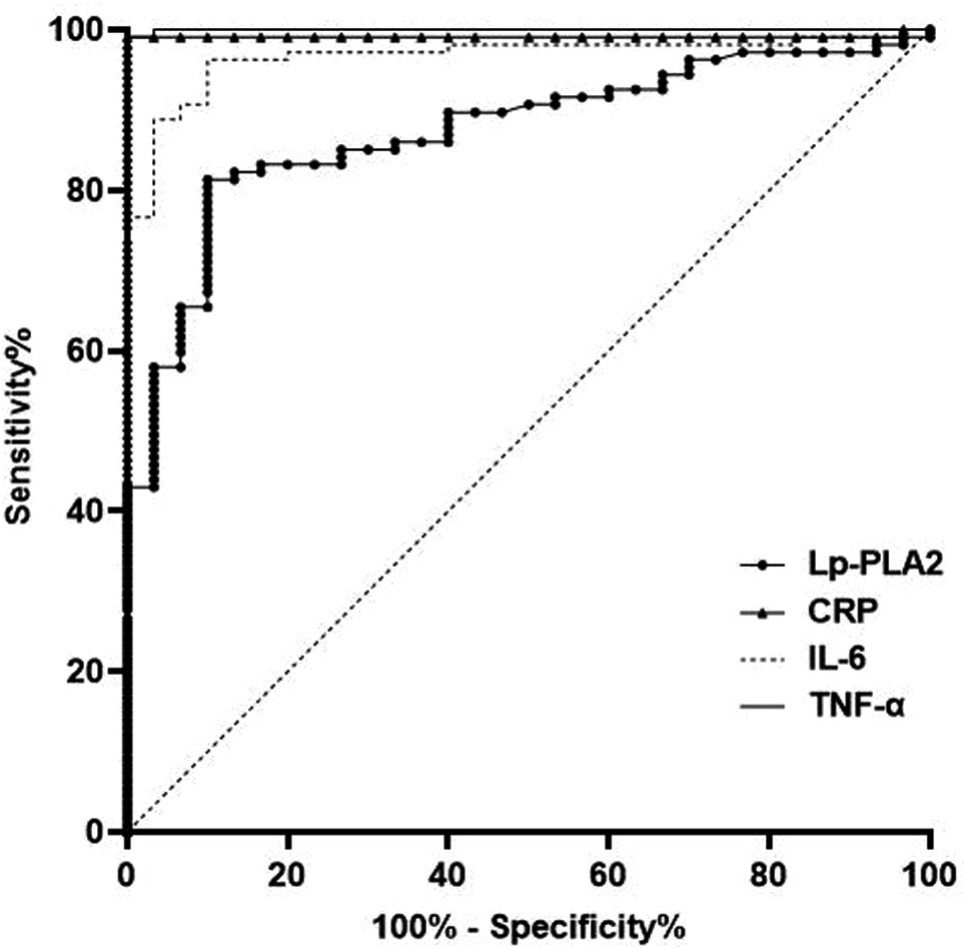
Predictive value of serum inflammatory factors in pregnant women with HDP.

The AUC of serum Lp-PLA2, CRP, IL-6 and TNF-α levels for predicting HDP were 0.873, 0.991, 0.966 and 0.999, respectively (*P* < 0.05) (Table 3 and Figure 4).

**Table 3:** Predictive value of serum inflammatory factors in pregnant women with HDP.

Index	AUC	95% CI		Std. Error	*P*-value	Youden index
		Lower limit	Upper limit			
Lp-PLA2	0.873	0.811	0.935	0.031	<0.0001	0.713
CRP	0.991	0.973	1.000	0.009	<0.0001	0.990
IL-6	0.966	0.937	0.996	0.014	<0.0001	0.862
TNF-α	0.999	0.998	1.000	0.001	<0.0001	0.991

## Discussion

At present, clinically, it is generally believed that the occurrence of HDP is related to heredity, immunity, nutritional deficiency, inflammatory reaction, vascular endothelial damage and other factors, and its occurrence and development are affected by environmental and psychological factors (7). The progress of HDP is closely related to the life and health of mother and infant, and the disease has gradually received attention in the field of pregnancy complications. Therefore, it is clinically necessary to judge the severity of HDP by monitoring serum indicators, and then improve the maternal and infant outcomes.

In this study, the levels of serum TC, TG and LDL-C increased with the progression of HDP, while the level of serum HDL-C decreased with the progression of HDP. This indicated that dyslipidemia may increase the risk of HDP, and it is closely related to the development of HDP. TC is the precursor of bile acids, steroid hormones and other substances, which is closely related to atherosclerosis and cardiovascular diseases. The main function of TG is to supply and store energy, and it can also fix and protect internal organs. LDL-C is a risk factor for vascular endothelial injury, and it can cause atherosclerosis. HDL-C is a vascular protective factor, which can remove lipids from blood vessels. In pregnant women with HDP, with the aggravation of the disease, the levels of TC, TG, LDL-C with vascular destruction increased, while the level of HDL-C with vascular protection decreased (8). High levels of TC and TG will lead to the decrease of prostacyclin synthesis in endothelial cells, resulting in imbalance between thromboxane A2 and prostacyclin, and the spasm of blood vessels, which will lead to the occurrence of preeclampsia (9). LDL-C is easily oxidized, and participates in the formation of atherosclerotic plaque, damages smooth muscle cells and endothelial cells, increases the permeability of vascular endothelial cells, reduces arterial lumen and increases peripheral resistance, which leads to the increase of blood pressure (10). The decrease of HDL-C may weaken the protective mechanism against atherosclerosis, inhibit the anti-lipid accumulation, leading to an increase in blood lipid peroxide, which directly damages the vascular endothelial cells, which will aggravate the severity of preeclampsia (11). In addition, estrogen, as a substance that reduces the lipase activity of liver endothelial cells, plays an inhibitory role in the absorption of HDL-C by the liver. Compared with normal pregnant women, pregnant women with HDP have less estrogen secretion and lower HDL-C release, which leads to compensatory responses and increases in serum TC, TG and LDL-C levels, further affects vascular endothelial cell functions and aggravates atherosclerosis (12). Elevated blood pressure can promote the formation of acute atherosclerosis, which in turn can promote the contraction of blood vessels, resulting in thickening and hardening of blood vessel walls, slow blood flow, obstruction of blood supply and oxygen supply to placenta, resulting in further increase of blood pressure, forming a vicious circle (13). The disorder of lipid metabolism in pregnant women with HDP can aggravate atherosclerosis, enhance oxidative stress, increase the production of peroxide products and toxic substances, and then damage vascular endothelial cells (14). At the same time, abnormal blood lipid levels can reduce the synthesis of nitric oxide, resulting in vasoconstriction and relaxation disorders, showing systemic arteriolar spasm and abnormally high blood pressure, which provides conditions for the disease progression of HDP (15).

We also found that the levels of serum Lp-PLA2, CRP, IL-6 and TNF-α were positively correlated with the severity of HDP. Lp-PLA2 can promote the release of inflammatory mediators, activate inflammatory response and damage vascular endothelial cells, which is an independent risk factor for preeclampsia. The possible mechanisms of Lp-PLA2 inducing HDP are as follows: ① Lp-PLA2 can combine apolipoprotein B with low density lipoprotein, causing lipid metabolism disorder, and then leading to atherosclerosis of uterine and placental arteries, resulting in decreased placental function, leading to the occurrence of HDP. ② Lp-PLA2 can hydrolysis and oxidation of lecithin, and produce strong pro-inflammatory mediators such as free fatty acids and lysolecithin. By activating cytokines and adhesion factors, vascular endothelial cells become dysfunctional, thereby increasing blood pressure. ③ Lp-PLA2 promotes the release of inflammatory mediators, collagen deposition and fibroblast proliferation in inflammatory reaction sites, and platelet aggregation, which plays an anti-inflammatory and anti-atherosclerosis role and promotes the progress of HDP (16–18). As a marker of inflammatory reaction in the body, CRP can activate platelet activity, inhibit platelet activating factor, promote platelet release of arachidonic acid and platelet aggregation, and inhibit the binding of platelet activating factor and neutrophils, which plays a role in regulating inflammatory reaction (19). CRP can not only aggravates the inflammatory reaction, but also reduces the sensitivity of tissue cells to insulin, and play a certain role in lipid metabolism disorder (20). There is inflammation in artery wall of pregnant women with HDP, and there are many trophoblast cells in blood from placenta, which can combine with antibodies in maternal blood to form immune complexes. CRP can combine with immune complex, activate the complement system of the body, produce a large amount of terminal reaction protein deposition, resulting in vascular endothelial cell damage, increase the production of vasoconstrictor substances, and then cause contraction and spasm of small blood vessels in the whole body, and increase blood pressure (21). The body of pregnant women with HDP is in a state of oxidative stress, which can promote neutrophil infiltration, release of a variety of proteases, inhibit vascular endothelial function, and lead to the increase of IL-6 and TNF-α levels (22). IL-6 can maintain chronic inflammation and promote the cascade amplification of inflammation. At the same time, IL-6 can interact with a variety of cytokines and induce with TNF-α, IL-1 and other inflammatory factors in the body (23). High levels of IL-6 can damage vascular endothelial cells, increase vascular permeability, interfere with vasoconstriction and relaxation, and lead to an increase in blood pressure, eventually leads to a series of clinical manifestations of preeclampsia (24). The increase of TNF-α can directly damage the vascular endothelium by releasing oxygen free radicals, causing systemic arteriole spasm, resulting in lumen stenosis and increased peripheral resistance, thereby exacerbating the progression of HDP (25). In pregnant women with HDP, TNF-α can promote the formation of neovascularization and thrombosis, inhibit the activities of lipolytic acid and lipoprotein lipic acids, which makes it difficult for lipids to dissolve, and then deposits on the wall of blood vessels, resulting in an increase in blood pressure (26).

In addition, ROC was used for further analysis in this study. The results showed that the AUC of serum TC, TG, HDL-C and LDL-C levels for predicting HDP were 0.759, 0.854, 0.770 and 0.785, respectively. The AUC of serum Lp-PLA2, CRP, IL-6 and TNF-α levels for predicting HDP were 0.873, 0.991, 0.966 and 0.999, respectively. This results further confirmed that the levels of blood lipid and serum inflammatory factor are closely related to HDP, which has certain value in predicting the occurrence and development of HDP. This is helpful to judge the occurrence of HDP and evaluate the status of HDP.

## Conclusion

To sum up, the levels of blood lipid and serum inflammatory factor are closely related to HDP, which has certain value in predicting the occurrence and development of HDP. We believe that dietary control and lifestyle changes can control blood lipids and reduce inflammation, and drug treatment can be used when necessary, which is conducive to improving the pathological changes of HDP. The sample size of this study is small, and a large number of clinical samples are still needed to provide further theoretical basis in the future, and it is also necessary to further explore the specific pathogenesis of HDP.

## Data Availability

The original contributions presented in the study are included in the article/Supplementary Material, further inquiries can be directed to the corresponding author/s.
